# Diagnosis and management of Achilles tendon ailments: the Scottish mist

**DOI:** 10.1186/s13018-024-04560-y

**Published:** 2024-02-09

**Authors:** Nicola Maffulli, John B. King, Filippo Migliorini, Otto Chan, Nat Padhiar, Filippo Spiezia

**Affiliations:** 1 Department of Trauma and Orthopaedic Surgery, Faculty of Medicine and Psychology, University La Sapienza, 00185 Rome, Italy; 2 School of Pharmacy and Bioengineering, Faculty of Medicine, Keele University, Stoke On Trent, ST4 7QB UK; 3 Centre for Sports and Exercise Medicine, Barts and the London School of Medicine and Dentistry, Mile End Hospital, Queen Mary University of London, London, E1 4DG England; 4 Department of Orthopaedic, Trauma, and Reconstructive Surgery, RWTH University Medical Centre, Pauwelsstraße 30, 52074 Aachen, Germany; 5 Department of Orthopedics and Trauma Surgery, Academic Hospital of Bolzano (SABES-ASDAA), Teaching Hospital of the Paracelsus Medical University, 39100 Bolzano, Italy; 6 Department of Imaging, The London Independent Hospital, London, E1 4DG England; 7 Department of Trauma Surgery and Orthopaedics, Hospital San Carlo, Potenza, Italy

**Keywords:** Achilles tendon, Tendinopathy, Physiopathology

## Abstract

The diagnosis and management of Achilles tendon ailments continue to be widely discussed by the scientific community. Also, the nomenclature used to describe the tendinopathic lesion in patients changed over the last decades together with the evolution in the knowledge of the physiopathology of Achilles tendinopathy, and unfortunately, through ignorance and possibly laziness, confusion still abounds. To emerge from these foggy paths, some clarifications are still necessary. The present Editorial tries to clarify some of these issues.

The role of high-resolution real-time ultrasound for the diagnosis and management of Achilles tendon ailments was put forward in the late 1980s [1], and only now it transpires that ultrasonography can be a major ally in this field [2].

We are nearly 2024 and learned colleagues still use the term 'tendinosis’ to describe the clinical syndrome of tendinopathy [2]. We pointed out in that tail end of the last millennium that the diagnosis of tendinosis can only be formulated based on histopathological analysis [3]. In addition, the present scientific evidence, as opposed to common parlance, shows that the essential lesion of tendinopathy is not degenerative, but it is a failed healing response [4] and that the role of inflammation in these lesions is being re-evaluated [5–7]. Even highly qualified orthopaedic surgeons, sports and exercise physicians, and physical medicine and rehabilitation specialists still use the terminology of 'Haglund's heel', 'Haglund's disease', 'Haglund's lesion', 'Haglund's syndrome', or similar, to indicate insertional tendinopathy of the Achilles tendon, at times associated with calcific deposits [8]. To avoid misunderstanding, we suggested more than a decade ago that such an eponym should be abandoned in favour of a more descriptive and factual term, namely (calcific) insertional tendinopathy [3, 5].

Many authors still report an imaging appearance of 'partial tears' of the Achilles tendon. At ultrasonography and MRI scanning, often imaging specialists report that 'a partial tear' of the Achilles tendon is present. Such lesions are, in clinical surgical practice, exceedingly rare. Also, the term ‘partial tears’ conveys the wrong impression to the patients and raises in their minds the spectre of an actual Achilles tendon rupture. At surgery, these areas which our imaging colleagues may name ‘partial tears’ are just an array of inhomogeneous tendinous tissue. There is no evidence of a tear at macroscopic and, above all, histopathological examination. In some instances, an actual intratendinous tear can be visualized [9], but it is present in only about 5% of patients with signs and symptoms of Achilles tendinopathy. These patients typically present with a history of sudden onset localised pain and the ability to train but not reach maximal loading, and especially being unable to sprint, which is especially prevalent in elite male athletes with a history of high-impact pain. We stress, however, that this is an uncommon presentation [9]. Finally, while imaging does play an important role in corroborating a clinical diagnosis of tendinopathy, repeated imaging is not indicated to monitor such patients [10]. Even in the presence of amelioration of symptoms, the appearance of the tendon may remain altered for a long while, and possibly forever, indicating a possible dissociation between tendon morphology and architecture and symptoms [10, 11].

Achilles tendon pathology is common and nevertheless is complex, with many facets. Management is not fully codified, and no sound evidence-based algorithms are available.

Let’s keep up the fight to inject some science into this field!

## Data Availability

The datasets generated during and/or analysed during the current study are available throughout the manuscript.
